# Muscle strength recovery after total hip arthroplasty and its impact on quality of life: findings from a Romanian cohort

**DOI:** 10.3389/fresc.2026.1760961

**Published:** 2026-03-30

**Authors:** Marjan Mihajlov, Alexandru Lisias Dimitriu, Monica Georgiana Roman, Elisa Georgiana Popescu, Eduard Cătălin Georgescu, Răzvan Ene, Dragoș Ene

**Affiliations:** 1 Department of Rehabilitation, Clinical Emergency Hospital of Bucharest, Bucharest, Romania; 2 Department of Orthopaedics and Traumatology, Clinical Emergency Hospital of Bucharest, Bucharest, Romania; 3 “Carol Davila” University of Medicine and Pharmacy, Bucharest, Romania; 4 Department of Surgery, “Carol Davila” University of Medicine and Pharmacy, Bucharest, Romania

**Keywords:** hip abduction, muscle strength, peak force, quality of life, rehabilitation, total hip arthroplasty

## Abstract

**Background:**

Musculoskeletal disorders represent a substantial global health burden, often leading to pain, functional impairment, and reduced quality of life. Total hip arthroplasty (THA) remains a key intervention for advanced hip pathology, yet postoperative recovery depends heavily on targeted rehabilitation. This study examined muscle strength progression and quality-of-life outcomes following a structured rehabilitation program after THA.

**Methods:**

Patients who underwent primary THA were enrolled in a structured postoperative physiotherapy protocol. Muscle strength (abduction, extension, and flexion peak force) was quantified using a validated digital dynamometer (ActivForce 2). Quality of life was evaluated using the Hip Disability and Osteoarthritis Outcome Score (HOOS), including the Quality-of-Life subscale (HOOS_QL). Assessments occurred at discharge (T1), six weeks post-discharge (T2), and twelve weeks postoperatively (T3).

**Results:**

Statistically significant longitudinal improvements in muscle strength were observed across all movement planes between T1–T2 and T2–T3 (*p* < 0.001). HOOS_QL scores showed a parallel increase over the same intervals. In regression analysis, hip extension peak force at T3 emerged as the only significant individual predictor of quality-of-life outcomes (*p* = 0.025).

**Conclusion:**

Participation in a structured postoperative rehabilitation program was associated with measurable improvements in muscle strength and patient-reported quality of life following THA. Hip extension strength was identified as an individual predictor of quality-of-life outcomes; however, the overall regression model demonstrated modest explanatory capacity, suggesting that this association warrants confirmation in larger controlled studies.

## 1 Introduction

Total hip arthroplasty (THA) is one of the most commonly performed procedures in contemporary orthopaedics and remains the definitive treatment for end-stage hip osteoarthritis. It provides substantial pain relief, restores mobility, and significantly improves overall quality of life.

Global data indicate a continuous rise in THA volume (1), a trend mirrored in Romania, where the annual incidence of primary hip arthroplasty has increased markedly over the past 15 years, reaching 51.8 procedures per 100,000 inhabitants in 2015 (2).

Despite generally favorable outcomes, a relevant proportion of patients experience persistent postoperative limitations, including chronic pain and reduced social participation (3, 4). These findings underscore the need to optimize postoperative rehabilitation strategies to enhance long-term functional recovery. Restoration of periarticular muscle strength has emerged as a critical component of this process (5).

After THA, significant strength deficits are commonly observed in the operated limb, with asymmetrical loading patterns that may persist for prolonged periods, particularly in hip abductor muscles, even up to 24 months postoperatively (6). Peak force, representing the maximum force output during a specific movement, is a key metric of muscular performance and a widely used parameter in rehabilitation and sports medicine (7).

Recent gait biomechanics research indicates that hip contact forces and joint loading patterns during gait after total hip arthroplasty exhibit distinctive profiles, with altered force magnitudes and kinetics compared with healthy peers. Studies have shown that hip contact force peaks remain a key determinant of functional outcomes and relate to patient-reported outcomes post-THA, and vertical ground reaction force peaks demonstrate progressive adaptation in load acceptance over the postoperative period. Moreover, detailed kinetic analyses reveal persistent differences in hip joint moments and power between operated and non-operated limbs in the months following surgery, highlighting compensatory loading patterns and asymmetries during gait (8–10).

Persistent weakness—particularly of the hip abductors and extensors—has been associated with altered biomechanics, gait instability, and reduced functional performance (11).

Recovery trajectories differ across muscle groups: abductors recover the slowest, reaching approximately 70% of normal strength at six months and 76% at twelve months, whereas hip flexors and extensors reach 80%–85% of healthy controls within one year (5, 12, 13).

Evidence shows substantial improvements in abduction, flexion, and extension strength within 3–6 months postoperatively, with some studies reporting near-complete recovery of abductor strength (up to 99% of the contralateral limb) within six weeks and flexion strength surpassing the non-operated side at three months (14–16).

Progressive rehabilitation programs enable patients to increase resistance in exercises such as standing hip flexion, abduction, and extension beginning approximately 4–6 weeks after surgery.

Although progressive rehabilitation programs are widely implemented and have been shown to improve muscle strength and functional outcomes (17), considerable heterogeneity exists regarding optimal strategies and objective monitoring methods. Recent studies have emphasized the value of quantitative biomechanical assessment tools in evaluating rehabilitation quality (18–20). However, despite increasing interest in postoperative rehabilitation outcomes, prospective longitudinal studies integrating objective peak force measurements with patient-reported quality-of-life outcomes remain relatively limited and methodologically heterogeneous, particularly in regional healthcare contexts (6).

Despite extensive literature on postoperative rehabilitation following THA, few longitudinal studies have simultaneously quantified objective peak muscle strength using digital dynamometry and examined its association with patient-reported quality-of-life outcomes across structured rehabilitation stages. Moreover, evidence from Eastern European cohorts remains limited.

The present study aimed to evaluate longitudinal changes in hip muscle peak force across three standardized rehabilitation stages and to explore the relationship between specific strength parameters and self-perceived quality of life in a Romanian cohort undergoing primary THA.

## 2 Methods

### 2.1 Study design

This study was designed as a prospective single-arm repeated-measures investigation aimed at examining longitudinal changes in muscle strength and self-reported quality of life following participation in a structured postoperative rehabilitation program after total hip arthroplasty (THA).

The independent variable was time, operationalized through three predefined assessment stages corresponding to rehabilitation progression. The dependent variables consisted of continuous quantitative measures of peak muscle strength of the hip abductors (HAS), extensors (HES), and flexors (HFS), as well as patient-reported quality of life.

Assessments were conducted at three time points:

T1: hospital discharge (baseline postoperative evaluation);
T2: six weeks post-discharge, when patients achieved independent ambulation;
T3: twelve weeks postoperatively, corresponding to completion of the structured rehabilitation program.

All participants underwent the same rehabilitation protocol and served as their own controls, allowing for within-subject longitudinal comparisons across the three assessment stages.

### 2.2 Participants

A total of 41 patients who underwent primary total hip arthroplasty at Euroclinic Hospital S.A., a nationally accredited medical center, were enrolled in the study. Participants were recruited prospectively between 1 October 2022 and 10 June 2023 from patients undergoing primary total hip arthroplasty (THA) at Euroclinic Hospital S.A. All patients who met the predefined inclusion criteria during this period were invited to participate in the study. All participants completed the postoperative rehabilitation program under the supervision of an experienced orthopaedic surgeon with over 25 years of clinical expertise in lower-limb trauma and joint reconstruction.

#### 2.2.1 Inclusion criteria

Participants were eligible for inclusion if they had undergone primary total hip arthroplasty (THA), were between 18 and 60 years of age, and provided voluntary written informed consent to participate in the study.

#### 2.2.2 Exclusion criteria

Patients were excluded if they experienced major postoperative complications such as hemarthrosis, periprosthetic fracture, or joint infection, or if they presented with conditions likely to affect long-term recovery, including neuropraxia. Additional exclusion criteria included any medical issue requiring hospital readmission within 90 days after surgery, as well as pre-existing musculoskeletal disorders of the lower limbs or spine that could impair mobility.

The upper age limit of 60 years was selected to reduce confounding factors related to advanced frailty, severe multimorbidity, and age-related sarcopenia, thereby ensuring a more functionally homogeneous cohort. In this population, common surgical indications included secondary osteoarthritis, femoral head osteonecrosis, post-traumatic degeneration, and developmental dysplasia of the hip.

### 2.3 Sample size calculation

The required sample size was calculated using G*Power version 3.1.9.7 based on a repeated-measures ANOVA design with one within-subject factor (three time points). A moderate expected correlation between repeated measures (r = 0.50) was assumed, consistent with short-term longitudinal strength assessments in postoperative rehabilitation studies.

The anticipated mean difference between time points was set at 1.5 units with an estimated standard deviation of 1.75, derived from previously published reports on hip muscle strength recovery following THA. These assumptions corresponded to an effect size f of approximately 0.30–0.35.

With *α* = 0.05 and power (1−*β*) = 0.80, the minimum required sample size was estimated at 35 participants. An additional 15% was considered to account for potential attrition.

Post hoc power analysis was not performed, as effect sizes and confidence intervals are reported directly in the Results section.

### 2.4 Instruments

Muscle strength (peak force) of the hip abductors, flexors, and extensors on the operated limb was assessed in abduction, extension, and flexion using the ActivForce Digital Dynamometer (AF2; Activbody, San Diego, CA). This hand-held dynamometer (HHD) is a validated device for monitoring exercise performance and quantifying muscle strength. Evidence indicates that the AF2 demonstrates comparable intra- and inter-tester reliability and criterion validity to the microFET2, with intraclass correlation coefficients (ICC) ranging from 0.85 to 0.99 across multiple movement patterns (21). Peak force values were recorded in Newtons (N).

All muscle strength assessments were performed by the same examiner to ensure measurement consistency.

#### 2.4.1 Abduction testing

Abductor strength was assessed with the patient standing upright against a fixed surface where the dynamometer was anchored. The hip and knee were positioned in neutral extension. The dynamometer strap was placed around the distal femur and stabilized proximally, approximately two finger-widths above the lateral femoral condyle. The examiner provided pelvic stabilization from behind. Patients were instructed to exert maximal effort— “push as hard and as fast as possible”—during a three-second isometric contraction following a verbal countdown (“3–2–1—start! Push, push, push—and relax!”). Key reliability criteria included maintaining pelvic stability, avoiding lumbar extension, and preventing pelvic rotation.

#### 2.4.2 Flexion testing

Hip flexor strength was evaluated in a seated position, with both thighs fully supported by the examination table. The dynamometer strap was anchored beneath the table, and the same three-second maximal isometric contraction and verbal countdown were employed. The examiner monitored posture to prevent compensatory activation of the rectus femoris or sartorius muscles.

#### 2.4.3 Extension testing

Hip extension strength was assessed with the participant positioned in a prone position on the examination table, with the pelvis stabilized manually by the examiner to prevent lumbar compensation. The tested limb was maintained in neutral hip rotation and full knee extension. The dynamometer strap was secured around the distal posterior thigh, just proximal to the popliteal fossa, and anchored to a fixed structure to ensure resistance consistency. Participants were instructed to perform a maximal isometric contraction by extending the hip against the strap resistance for 3 s, following the standardized verbal countdown (“3–2–1–start”). Care was taken to avoid compensatory lumbar extension or pelvic rotation. Each contraction was performed under identical conditions across all assessment time points.

Each movement (abduction, flexion, and extension) was tested in three consecutive maximal isometric trials. A familiarization trial was performed prior to formal testing to ensure correct technique and patient understanding. A rest interval of 30–60 s was allowed between trials to minimize fatigue. The highest peak force value obtained from the three trials was used for statistical analysis (Table 1).

**Table 1:** Exercise types for neuromuscular activation – key characteristics (text description for manuscript).

Key Principle	Description
Neuromuscular activation	Fundamental component of restoring muscle function.
Effect of pain	Pain inhibits muscle activation; reduction of pain enhances performance.
Types of interventions	May include assisted/self-assisted movements, eccentric or isometric contractions within controlled ranges.
Immobilization effects	Prolonged immobilization may require extended activation intervals and gradual reactivation.
Coordination	Sequential activation and coordinated contraction patterns are essential to correct functional imbalances.

The dynamometer was calibrated according to the manufacturer's specifications prior to the initiation of the study and periodically checked to ensure measurement accuracy throughout the data collection period. All assessments were performed by the same examiner to reduce inter-tester variability.

#### 2.4.4 Quality of life assessment

Self-perceived quality of life was measured using the Hip Disability and Osteoarthritis Outcome Score (HOOS), a validated instrument with strong psychometric properties in THA populations (22).

#### 2.4.5 Sociodemographic data

Sociodemographic and clinical characteristics were recorded via a 15-item structured questionnaire capturing age, sex, residential background (urban/rural), educational level, occupation, weight, height, date and type of THA (lateral or minimally invasive approach), healthcare setting (public or private), length of hospitalization, presence and duration of preoperative training, number of preoperative sessions, and comorbidities.

All evaluations were conducted in a standardized sequence and by the same evaluator to ensure internal consistency and minimize measurement bias.

### 2.5 Ethical considerations

The study was approved by the Euroclinic Hospital Ethics Committee (Approval No. 0006168, Institutional Registry No. 26/13). All procedures involving human participants were conducted in accordance with institutional guidelines and national ethical standards. Written informed consent was obtained from all participants on the day of hospital discharge.

### 2.6 Rehabilitation program

The postoperative rehabilitation program was initiated on the first day following surgery. Exercise selection was informed by clinical expertise and established rehabilitation guidelines (e.g., American Academy of Orthopaedic Surgeons; Royal Dutch Society for Physical Therapy). Early recovery followed standard THA protocols, after which patient-specific modifications were introduced according to biomechanical parameters and individual functional needs.

The rehabilitation protocol comprised three progressive phases:

1. Acute Phase (0–7 days)
  Sessions were conducted daily during hospitalization (1–2 sessions/day, approximately 20–30 min each). Interventions included respiratory exercises, ankle pumps, isometric quadriceps and gluteal contractions, assisted hip flexion within safe range, and early gait training with assistive devices. Intensity was low and pain-limited, focusing on neuromuscular activation and prevention of deconditioning.
2. Subacute Phase (2–6 weeks)
  Patients attended supervised physiotherapy sessions 3 times per week (45–60 min/session). Exercises included active hip abduction, extension, and flexion against body weight or elastic resistance bands, sit-to-stand training, step exercises, balance training, and progressive gait re-education. Intensity was gradually increased toward moderate levels (approximately 60%–70% of estimated one-repetition maximum or perceived exertion 4–6/10). Progression criteria included absence of pain exacerbation, stable gait pattern, and ability to complete 3 sets of 10–12 repetitions without compensatory movement.
3. Maintenance Phase (6–12 weeks)
  Supervised sessions continued 2–3 times per week, supplemented by a home exercise program. Resistance training intensity progressed to approximately 70%–85% of estimated one-repetition maximum, targeting 8–12 repetitions per set. Exercises included resisted hip abduction, extension, and flexion, functional strengthening (lunges, step-ups), proprioceptive drills, and endurance walking. Progression was based on tolerance, absence of compensatory biomechanics, and achievement of functional milestones.

Throughout all phases, exercise progression was individualized based on clinical evaluation, pain response, and functional performance.

#### 2.6.1 Neuromuscular activation principles

The primary priority in restoring muscle function was neuromuscular activation, particularly correct sequencing, timing, and intensity of muscle recruitment. Based on individual neuromuscular patterns, exercises were selected to target specific deficits, support postural control, or improve endurance through either direct activation (active contraction) or indirect facilitation (pain-reduction strategies).

In cases of immobilization-related weakness or nerve injury, supplementary modalities such as sensory feedback, electrical stimulation, or biofeedback were incorporated.

#### 2.6.2 Strengthening exercises

Strengthening strategies relied on body-weight movements or external resistance (Table 2), with progression based on established recommendations for hypertrophy and functional rehabilitation.

**Table 2:** Characteristics of therapeutic strengthening exercises – key points - progressive overload is essential for inducing hypertrophy.

Key Concept	Description
Progressive overload	Essential for promoting muscular hypertrophy and strength adaptation.
Body-weight resistance	Sufficient for achieving basic toning and foundational strength goals.
Exercise specificity	Must account for range of motion, contraction duration, and functional demands of the targeted movement.
Gradual load progression	Load should be increased progressively after adequate adaptation.

Loads equivalent to 75%–85% of the one-repetition maximum (1RM) were applied, typically targeting 8–12 repetitions per set, in accordance with ACSM recommendations. Progression was achieved primarily by increasing the number of sets before modifying resistance or contraction time.

The strengthening program followed the framework proposed by Dunleavy & Slowik (23), which categorizes exercises based on the targeted tissue and muscular role (local stabilizers, global stabilizers, global mobilisers).

### 2.7 Statistics

Data analysis was performed using IBM SPSS Statistics version 23.0 for Windows (IBM Corp., Armonk, NY, USA) (24). Normality was assessed using the Shapiro–Wilk test, which is recommended for small sample sizes (*N* < 50).

As several muscle strength variables demonstrated deviations from normality across assessment stages, longitudinal within-subject comparisons were conducted using the non-parametric Wilcoxon signed-rank test.

Descriptive statistics included means and standard deviations. Associations between variables were evaluated using Pearson correlation coefficients when normality assumptions were satisfied. Predictive relationships were examined using hierarchical multiple regression analysis.

Statistical significance was set at *p* < 0.05 with a 95% confidence interval.

## 3 Results

**Figure 1:**
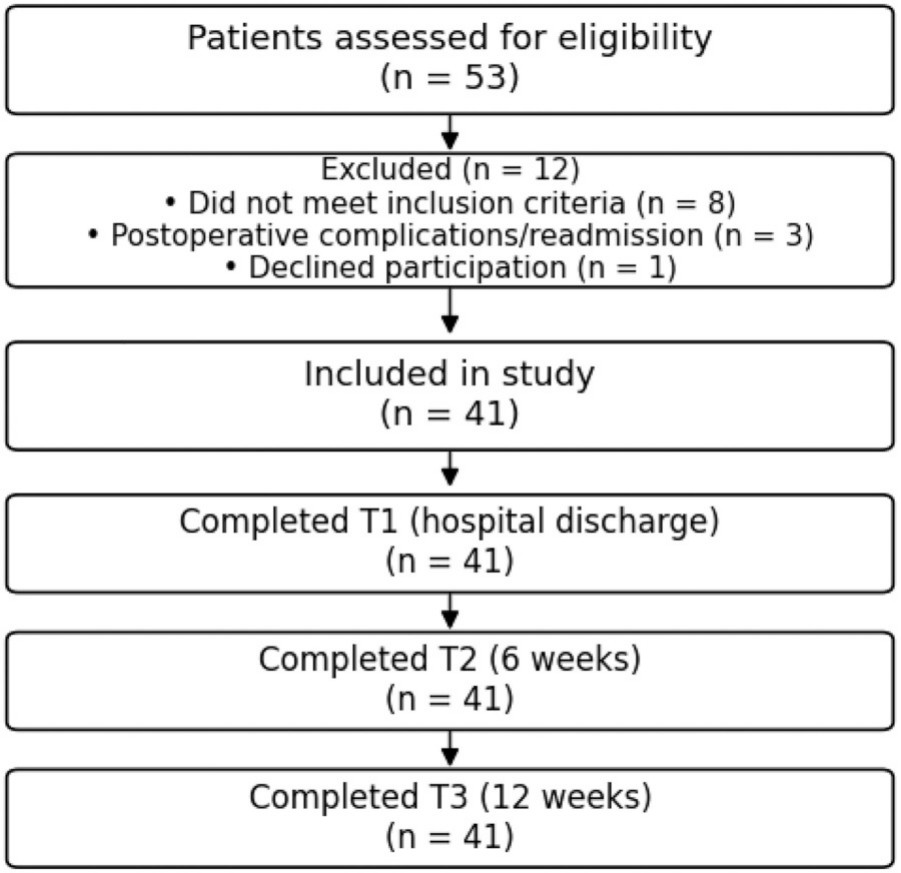
Participant flow diagram. Patients assessed for eligibility (*n* = 53), excluded (*n* = 12), included (*n* = 41), and completed assessments at T1 (hospital discharge), T2 (6 weeks), and T3 (12 weeks) with no dropouts.

A flow diagram summarizing patient screening, exclusions, and follow-up across all assessment time points is presented in Figure 1.

### 3.1 Descriptive statistics

The study included 41 participants, of whom 27 were male and 14 were female. Participant ages ranged from 28 to 56 years (M = 46.48, SD = 6.42), and body weight varied between 46 and 105 kg (M = 79.58, SD = 14.45). Seventeen participants underwent surgery on the left hip, while twenty-four had surgery on the right. A lateral surgical approach was used in 19 cases, whereas 22 procedures were performed using a minimally invasive approach. Only four participants had engaged in preoperative physiotherapy.

At hospital discharge (T1), descriptive statistics were calculated for peak muscle strength in hip abduction, extension, and flexion, as well as for self-perceived quality of life. These values are summarized in Table 3.

**Table 3:** Descriptive statistics for hip abductor, extensor, and flexor peak force (N) and perceived quality of life at hospital discharge (T1).

Variable	N	Minimum	Maximum	Mean	SD
HAS peak force₁	41	1.01	5.12	2.77	1.08
HES peak force₁	41	0.95	6.19	3.16	1.35
HFS peak force₁	41	1.47	9.35	3.18	1.42
HOOS_QL₁	41	12	20	16.68	2.41

HAS, Hip Abduction Strength; HES, Hip Extension Strength; HFS, Hip Flexion Strength; HOOS_QL, Hip Disability and Osteoarthritis Outcome Score (Quality of Life subscale).

### 3.2 Muscle strength across rehabilitation stages

To determine whether muscle strength improved across the three evaluation stages (T1, T2, and T3), paired comparisons were conducted using the Wilcoxon signed-rank test. Significant increases were observed for all muscle groups in both time intervals (Table 5).

For hip abduction strength (HAS), performance improved significantly from T1 to T2 (Z = −5.48, *p* < .001, r = .86) and from T2 to T3 (Z = −4.89, *p* < .001, r = .76).

Similarly, hip extension strength (HES) showed significant gains from T1 to T2 (Z = −5.46, *p* < .001, r = .85) and from T2 to T3 (Z = −4.64, *p* < .001, r = .72).

Hip flexion strength (HFS) also increased significantly between T1 and T2 (Z = −5.57, *p* < .001, r = .87) and between T2 and T3 (Z = −4.26, *p* < .001, r = .67).

All effect sizes were large, indicating robust improvements in peak muscular strength and supporting a positive longitudinal improvement in muscle strength across all three assessment points.

Descriptive statistics for each muscle group (T1, T2, T3) are presented in Table 4.

**Table 4:** Descriptive statistics for peak muscle strength (N) across rehabilitation stages (T1, T2, T3).

Variable	Stage	N	Mean	SD	Minimum	Maximum
HAS	T1	41	2.77	1.08	1.01	5.12
	T2	41	6.25	2.32	1.83	11.45
	T3	41	9.65	3.40	3.16	18.97
HES	T1	41	3.16	1.35	0.95	6.19
	T2	41	6.28	2.60	1.12	11.75
	T3	41	9.46	3.17	3.25	18.84
HFS	T1	41	3.18	1.42	1.47	9.35
	T2	41	7.51	3.89	1.68	22.02
	T3	41	10.43	3.15	3.47	18.00

HAS, Hip Abduction Strength; HES, Hip Extension Strength; HFS, Hip Flexion Strength.

T1=hospital discharge; T2=6 weeks post-discharge; T3=end of rehabilitation (12 weeks).

**Table 5:** Wilcoxon signed-rank test – evolution of muscle strength between stages (T1–T2–T3).

Muscle Group	Comparison (Stage)	Z-value	*p*-value	Effect size (r)	Significance
HAS (Hip Abduction Strength)	T2−T1	−5.48	<.001	.86	***
	T3−T2	−4.89	<.001	.76	***
HES (Hip Extension Strength)	T2−T1	−5.46	<.001	.85	***
	T3−T2	−4.64	<.001	.72	***
HFS (Hip Flexion Strength)	T2−T1	−5.57	<.001	.87	***
	T3−T2	−4.26	<.001	.67	***

All comparisons indicated significant improvement in muscle strength between consecutive stages.

Peak force values expressed in Newtons (N).

*** *p* < 0.001.

### 3.3 Self-perceived quality of life

To evaluate changes in self-perceived quality of life during the rehabilitation program, the Wilcoxon signed-rank test was applied to paired HOOS_QL scores between T1 and T3 (Table 6).

**Table 6:** Wilcoxon signed-rank test for HOOS_QL improvement (T1–T3).

Comparison	Z	*p*-value	Significance
HOOS_QL T3 – HOOS_QL T1	−5.59	<.001	***

The improvement in HOOS_QL scores demonstrates strong statistical significance and supports the clinical relevance of the intervention.

***p < .001.

A clinically and statistically significant improvement was observed across all participants (Z = −5.59, *p* < .001), indicating substantial gains in both functional recovery and subjective well-being. These results confirm the positive impact of the structured rehabilitation program on patient-reported outcomes.

The improvement in quality-of-life scores supports the strong clinical relevance of the intervention, aligning with previous findings that structured physiotherapy reduces pain, enhances functional capacity, and improves overall health perception following total hip arthroplasty.

### 3.4 Relationship between peak force and quality of life

The associations between muscle strength parameters at 12 weeks (HAS, HES, HFS at T3) and self-perceived quality of life (HOOS_QL at T3) were examined using Pearson correlation and multiple regression analysis. Correlation coefficients are presented in Table 7.

**Table 7:** Pearson correlations between peak muscle strength (N) and self-perceived quality of life at T3.

Variable	HOOS_QL T3	HAS T3	HES T3	HFS T3
HOOS_QL T3	1	.092	.312*	−.051
HAS T3	.092	1	.715**	.574**
HES T3	.312*	.715**	1	.577**
HFS T3	−.051	.574**	.577**	1

*p < .05.

.**p < .01.

A significant positive correlation was observed only between hip extension strength (HES T3) and HOOS_QL (r = .31, *p* < .05), indicating that greater extension strength at 12 weeks is associated with better self-reported quality of life. No significant correlations were found for hip abduction (HAS T3) or hip flexion strength (HFS T3).

To further explore predictive relationships, a hierarchical multiple regression model was used.

Model 1, including age, weight, and height, was not significant [F(3, 37) = 1.58, *p* = .212], explaining 11.3% of the variance (R^2^ = .113).

Model 2, which added HAS, HES, and HFS peak force values, improved the predictive ability [F(6, 34) = 2.15, *p* = .073], accounting for 27.5% of the variance (R^2^ = .275; adjusted R^2^ = .147).

Within Model 2, hip extension peak force (HES T3) was the *only* significant predictor of HOOS_QL [*β* = .53, t = 2.34, *p* = .025; B = 0.55, 95% CI (0.07–1.03)]. Body weight showed a marginal negative trend (*p* = .055), while all other predictors were non-significant (*p* > .05).

The hierarchical regression analysis demonstrated that hip extension strength (HES T3) was the only significant predictor of self-perceived quality of life at 12 weeks (*β* = .53, *p* = .025). Although body weight showed a marginal negative trend (*p* = .055), all other predictors—including age, height, and peak abduction (HAS) and flexion strength (HFS)—were non-significant (*p* > .05).

The full model accounted for 27.5% of variance in HOOS_QL (R^2^ = .275; adjusted R^2^ = .147), indicating moderate predictive power appropriate to the sample size (*N* = 41). Multicollinearity diagnostics indicated acceptable tolerance values (>0.80) and low Variance Inflation Factor (VIF) values (all < 2.0), confirming the absence of problematic multicollinearity among predictors (HAS: VIF = 1.32; HES: VIF = 1.41; HFS: VIF = 1.35) (Table 8).

Although hip extension strength (HES T3) emerged as a statistically significant individual predictor, the overall regression model did not reach conventional statistical significance (*p* = .073) and demonstrated modest explanatory power (adjusted R^2^ = .147). Therefore, these findings should be interpreted cautiously and considered exploratory.

**Table 8:** Hierarchical regression predicting self-perceived quality of life (HOOS_QL T3).

Predictor	B	SE	*β*	t	p	95% CI
Constant	6.91	4.88	—	1.42	.166	[−3.00, 16.83]
Age	0.01	0.08	.03	0.17	.865	[−0.14, 0.17]
Weight	−0.07	0.04	−.32	−1.99	.055	[−0.15, 0.00]
Height	0.00	0.01	.05	0.31	.760	[−0.02, 0.04]
HAS T3	−0.02	0.23	−.02	−0.07	.942	[−0.49, 0.46]
HES T3	0.55	0.24	.53	2.34	.025	[0.07, 1.03]
HFS T3	−0.28	0.21	−.26	−1.30	.201	[−0.70, 0.15]

Dependent variable, HOOS_QL T3. *N* = 41. Peak force values expressed in Newtons (N).

## 4 Discussion

### 4.1 Theoretical and clinical relevance of the results

The findings of this study align closely with previously published international research on postoperative recovery following total hip arthroplasty (16, 21, 25).

The observed progressive improvements in hip abductor, extensor, and flexor strength over the 12-week rehabilitation period suggest that structured physiotherapy is associated with meaningful functional gains after THA. These results reinforce the concept that muscle strength recovery remains a central component of successful postoperative adaptation.

Importantly, objective quantification of muscle performance through digital dynamometry addresses limitations highlighted in earlier work. Boekesteijn et al. (26) emphasized that static radiographic or clinical assessments do not adequately capture functional performance during dynamic, weight-bearing activities. By incorporating peak force measurements, the present study provides reproducible biomechanical data that complement patient-reported outcomes and enhance clinical monitoring precision. The broader rehabilitation literature also supports the importance of structured postoperative programs. Costa et al. (27) reported consistent improvements in pain and functional outcomes after THA but noted the lack of consensus regarding optimal rehabilitation strategies. In this context, the present results suggest that strength-oriented, progressively structured rehabilitation—supported by objective measurement tools—may represent a clinically meaningful approach.

Furthermore, multicenter evidence indicates that patient comorbidities significantly influence early postoperative recovery (28), underscoring the need for personalized and closely supervised rehabilitation pathways.

Biomechanical considerations further contextualize these findings. Biotribological analyses demonstrate that implant loading and bearing surface behavior are strongly influenced by joint forces and movement quality (29). Persistent muscular weakness may therefore contribute to abnormal contact stresses, highlighting the clinical relevance of restoring symmetrical loading through targeted strengthening. This interpretation aligns with data showing that frailty, performance capacity, and functional independence modulate recovery trajectories in patients with THA-related complications (30), as well as with surgical reports suggesting that approach-related biomechanical factors influence postoperative stability and function (31).

Objective biomechanical assessment has also gained increasing support in recent literature. Vișan et al. (32) identified muscle deflection as a valid indicator of rehabilitation quality, reinforcing the value of quantifiable musculoskeletal parameters in evaluating functional recovery. Together, these observations support the integration of measurable strength metrics—such as peak force—into routine postoperative assessment frameworks.

Within the Romanian clinical context, structured and quantitatively driven postoperative rehabilitation research remains limited. By combining longitudinal strength measurements with quality-of-life evaluation, the present study contributes regional data and proposes a reproducible methodological model that may support evidence-based postoperative care.

Although hip extension strength emerged as an individual predictor of self-perceived quality of life, the overall regression model demonstrated modest explanatory capacity and did not reach conventional statistical significance. Accordingly, this association should be interpreted cautiously and considered exploratory rather than confirmatory.

The novelty of the present study lies in its longitudinal within-subject design integrating objective dynamometric peak force measurements with patient-reported quality-of-life outcomes across clearly defined rehabilitation stages. While numerous studies have examined functional recovery after THA, few have investigated the specific contribution of hip extension strength to perceived quality of life within a structured stage-based program. These findings therefore provide hypothesis-generating evidence that may help refine targeted rehabilitation priorities.

### 4.2 Strengths and limitations

The interpretation of the present findings should take into account several methodological limitations. First, the sample size was modest (*N* = 41), which may limit external validity and reduce the ability to detect smaller effect sizes.

Second, the non-randomized sampling approach introduces the possibility of selection bias. Furthermore, the same investigator acted as both therapist and evaluator, which may increase the risk of measurement bias. Although the use of independent blinded assessors would have further strengthened methodological rigor, a single trained examiner was intentionally maintained throughout the study to ensure procedural consistency and minimize inter-rater variability across repeated assessments. In addition, muscle strength was measured using a validated digital dynamometer with high reported reliability, thereby reducing subjective influence. Nevertheless, future studies should incorporate independent blinded evaluators to further limit potential bias.

Third, the absence of a control group (e.g., standard care or unsupervised rehabilitation) limits the ability to draw causal conclusions regarding the specific efficacy of the structured rehabilitation program. Although statistically significant longitudinal improvements were observed, it remains unclear to what extent these gains reflect the structured intervention vs. natural postoperative recovery. Future randomized controlled trials are needed to establish comparative effectiveness.

Additionally, the relatively young age distribution (mean 46.5 years) may reduce the generalizability of the findings to older primary osteoarthritis populations, who represent a substantial proportion of THA recipients. Functional recovery patterns and rehabilitation responsiveness may differ in elderly patients with higher levels of frailty or comorbidity. Therefore, extrapolation of these results to older cohorts should be undertaken cautiously. The predefined age range was selected to ensure a functionally homogeneous cohort and reduce confounding related to advanced frailty; however, this necessarily narrows external validity.

The use of objective digital dynamometry (ActivForce 2) represents a key strength, offering reliable and quantifiable measures of muscle function that surpass the limitations of subjective clinical assessment. This level of measurement precision strengthens the evaluation of functional gains.

Moreover, the integration of objective biomechanical data with patient-reported quality-of-life outcomes provides a comprehensive understanding of postoperative recovery. The consistency of measurement procedures and the longitudinal design further support the internal validity of the findings, enabling clinicians to monitor recovery trajectories and tailor rehabilitation progression more effectively.

### 4.3 Future directions and research needs

Future research on postoperative muscle strength recovery following THA should prioritize longitudinal study designs capable of capturing strength trajectories over extended periods and identifying predictors of sustained improvement.

Comparative trials are also warranted to evaluate the differential impact of specific rehabilitation modalities—such as resistance training, proprioceptive exercises, aquatic therapy, and neuromuscular stimulation—on both strength and functional outcomes.

Emerging evidence highlights the importance of genetic, biological, and biomechanical factors in explaining inter-individual variability in rehabilitation response. A deeper understanding of these determinants could support the development of personalized rehabilitation strategies tailored to each patient's physiological and functional profile.

There is also growing momentum toward integrating digital health technologies into postoperative care. Wearable sensors, tele-rehabilitation platforms, and real-time performance monitoring tools have the potential to optimize exercise intensity, improve patient adherence, and facilitate ongoing clinical feedback (5).

These technologies may play a transformative role in the future of rehabilitation delivery.

In summary, future investigations should further explore individualized and technology-assisted recovery pathways, assess their long-term effectiveness, and reinforce the evidence base for structured physiotherapy protocols aimed at optimizing functional outcomes and quality of life in patients undergoing THA.

## 5 Conclusion

Recovery of muscular strength appears to be closely associated with functional outcomes and quality of life following total hip arthroplasty. The present study observed significant and progressive improvements in hip abduction, extension, and flexion strength over the course of a structured, stage-based rehabilitation program. Early initiation and consistent participation in physiotherapy may contribute to optimizing biomechanical recovery and limiting compensatory dysfunction.

Hip extension peak force was associated with improved self-perceived quality of life; however, the predictive model demonstrated modest explanatory capacity and did not reach overall statistical significance, indicating that these findings should be interpreted cautiously and confirmed in larger controlled studies.

Overall, the results support the potential relevance of individualized, objectively monitored, and strength-focused rehabilitation strategies in facilitating recovery after THA, while acknowledging the need for further comparative research.

## Data Availability

The dataset contains sensitive clinical information and is subject to institutional and national data-protection regulations. For this reason, the raw data cannot be publicly shared or deposited in an open repository. Anonymized data may be made available upon reasonable request to the corresponding author: alexandru.dimitriu@umfcd.ro
